# Subtle Signals of Status on Social Network Sites: Evidence From China

**DOI:** 10.3389/fpsyg.2021.741602

**Published:** 2021-09-17

**Authors:** Wangshuai Wang, Tiantian Shao, Yanxi Yi, Shijiao Fang, Jingyi Song, Zucheng Yu

**Affiliations:** ^1^School of Management, Shanghai University of International Business and Economics, Shanghai, China; ^2^School of International Relations and Public Affairs, Shanghai International Studies University, Shanghai, China

**Keywords:** SNS, subtle signals, social status, perceived busyness, Chinese consumers

## Abstract

Social network sites (SNS) have been indispensable channels for people to access information, present themselves, and conduct commercial activities. Existing literature on online consumer behavior mainly focus on Western consumers and on explicit conspicuous signals. However, reports have shown that SNS users in China have exceeded 370 million, ranking the first in the world. Meanwhile, more and more consumers display status in more implicit ways nowadays. To fill these gaps, the present research was conducted to investigate the subtle signals of status for Chinese consumers on SNS. We proposed that frequent SNS posting leads to higher status perception among Chinese consumers. The psychological process of this effect is perceived busyness. These hypotheses received convergent support in a set of three studies. Study 1 used secondary data to preliminarily verify the positive correlation between SNS posting frequency and perceived social status. Studies 2A and 2B adopted the causal chain method to test the underlying mechanism of the effect, and to provide causal evidence for the entire relationship chain. Specifically, Study 2A examined how SNS posting frequency affects perceived busyness. Furthermore, Study 2B explored whether the differences in perceived busyness will affect social status perceptions. Implications of these findings and potential extensions in future are discussed.

## Introduction

Digital network, mobile and social network sites (SNS), have become an indispensable part of the daily lives of people all over the world. In January 2021, the population of the world has reached 7.83 billion and the number of SNS users is 4.2 billion. This figure has increased by 490 million in the past year with a year-on-year increase of more than 13%. The number of SNS users now accounts for more than 53% of the global population. On an average, more than 1.3 million new users join SNS every day which means about 155,000 new users join SNS per second (Sina Finance, 2021). Surprisingly, over one in five people use SNS in the world. SNS may be the biggest communication system in the last 15 years since it enabled people to connect with others and express their views on a global scale. The number of SNS users in China has reached 1.04 billion, with a penetration rate of 72%, far higher than the global rate of 49%. According to the report “digital 2020” that was jointly released by We Are Social (2020), there are 1.04 billion SNS users in China, accounting for a large proportion of the 3.8 billion people who use SNS around the globe. SNS users in China have increased by 15 million, and each user has an average of 9.3 accounts.

Due to the rapid development of SNS, many scholars have devoted themselves to this field. Researches have shown that SNS has been an indispensable part in the daily life of people. When people are immersed in SNS for a long time, their attitudes, behaviors, and living habits are deeply influenced by SNS (e.g., Heinonen, 2011; Bai and Hao, 2013; Yang et al., 2017; Abeza et al., 2020; Cao et al., 2021). SNS is one of the “best opportunities” for a brand to connect with potential consumers. It can make a deeper connection with consumers, gain the trust of consumers, and further guide consumer behaviors (Sajid, 2016). The research of Bellezza et al. (2017) shows that when the cultural background is different, the information conveyed to other users by SNS posting state is different. Some scholars have also found that compared with collectivist countries such as China and Thailand, individualistic countries such as the United Kingdom, the United States, and Australia are less likely to use SNS to make purchase decisions. In China and Thailand, SNS plays an important role in forming opinions (Goodrich and De Mooij, 2014). It is worth noting that current researches on online consumer behavior mainly focus on Western consumers, and the research into the characteristics of high social status users needs to be explored in the context of Chinese society.

Scholars have indicated that wealthy people present their social status by showing off their expensive luxury goods (Berger and Ward, 2010). Valsesia et al. (2020) found that under the premise of having a large number of followers, people with less followers have a positive impact on SNS users. Some scholars have also found that the subtle signals of perceived busyness on SNS can illustrate their social status. The simple self-expression on SNS has gradually been unable to satisfy users. Additionally, there is exaggerated information on the WeChat Moments of some users. An increasing number of users “show” their superiority on WeChat Moments to show their social status (Shen, 2020). Recent researches have demonstrated that users use WeChat Moments to show off their status, culture, connections, taste, etc. to further demonstrate their individual socioeconomic status, which can help them meet the personalized needs of the self-expression of the users (Shao, 2020). The majority of the current researches have to do with direct conspicuous consumption (e.g., Ordabayeva and Chandon, 2011; Wang et al., 2015; Gao et al., 2016), while lack of attention to subtle signals.

The research on conspicuous consumption has started ever since last century. Consumers can transmit the information about their social and economic status through a series of products (Ordabayeva and Chandon, 2011). Some scholars have shown that people present their social status by showing off their personal goods (Belk et al., 1982; Burroughs et al., 1991). Users with high social status usually play a guiding role on social media, that is, they can play the role of opinion leaders. Opinion leaders are individuals who convey a large amount of information on SNS and deliver the information to other users (Arndt, 1967). Individuals with high social influence or social status feel entitled to more than low-status individuals and can often influence the consumer behaviors of other people (Hu et al., 2016). The common social media marketing strategy is to identify a group of opinion leaders and let them share specific products or brands, since the products or brands shared by individuals with high social status are more likely to be trusted and accepted by the public (Saravanakumar and Sugantha Lakshmi, 2012; Valsesia et al., 2020). Existing literature on online consumer behaviors mainly put focus on Western consumers, whereas research concentrating on the characteristics of high social status users in the Chinese social context is still in its infancy. This study focuses on how the behavior of SNS users affects social status perception in the eyes of others in the context of Chinese society. We test the underlying mechanism of the effect. Furthermore, what is the psychological mechanism of the effect? Does this effect always exist? The current literature does not answer these questions.

The present research investigated the subtle signals of status for Chinese consumers on SNS. Our research mainly was concerned with the relation between SNS posting frequency and perceived social status among Chinese consumers. We proposed that frequent SNS posting leads to the higher status perception among Chinese consumers. The psychological process of this effect is perceived busyness. The user who has frequent SNS posting will be perceived as leisurely by others, which can be further inferred that they have a higher social status. These hypotheses received convergent support in both laboratory experiments and secondary data from the real situation. The present research takes the influence of cultural differences into consideration. In addition, the present research also makes a theoretical contribution to the literature on conspicuous consumption that further enriches the literature on subtle signals of status.

This research contributes greatly to the corresponding literature. Firstly, the research makes a theoretical contribution to the literature of SNS. Although previous researches have begun to study online consumer behavior (e.g., Williamson, 2010; Khan, 2017; Mieczkowski et al., 2020), this research explores the differences in SNS posting frequency to show different communication information. Users who have frequent SNS posting are often considered to spend more time on SNS, and so they are considered more at leisure. Conversely, users who have less frequent SNS posting are often considered to spend less time on SNS which means they are busier (Bellezza et al., 2017). Secondly, previous researches on online consumer behavior mainly focus on Western consumers (Williamson, 2010; Hajli, 2014; Abeza et al., 2017; Bellezza et al., 2017), while this research found that different level of busyness has different effects on social status in different cultural backgrounds. Bellezza et al. (2017) demonstrated that busy people are considered to have a higher social status in the United States. In China, we found that busy people are considered to have a lower social status than those who are comparatively less busy. This is because people must devote a lot of time to work in order to meet the basic needs of life, and so busy people have a lower social status (Li and Zhou, 2014).

Thirdly, previous researchers found that consumers can transmit the information of their social and economic status through explicit conspicuous signals (Belk et al., 1982; Burroughs et al., 1991; Ordabayeva and Chandon, 2011). An increasing number of consumers display status in more implicit ways. The research investigates how users can efficiently use SNS to display status, in a word, how SNS users in China can improve their social status. The research has made further explorations that the busy signals released on social media platforms can play the role of a mediator. The research also explored whether the differences in perceived busyness would affect social status perceptions. Besides, a detailed study about the subtle signal show-off on emerging social media has been made, thereby refining the research topics in this field.

## Theoretical Background and Hypothesis Development

### Social Network Sites and Perceived Busyness

Social network sites are Internet applications based on the ideas and technologies of Web 2.0 (Internet 2.0). Users can use SNS for creation, emotional communication, and information sharing (Abeza et al., 2017). Some scholars also defined SNS as an online platform that allows users to create, share, and exchange information. The content carried by SNS includes texts, images, audio, and video (Mieczkowski et al., 2020). The emergence of social media is conducive for people to access information, present themselves, and conduct commercial activities (Khan, 2017). In other words, SNS uses a series of communication software for information exchange, and users can share their views and opinions on the SNS. Information transmission on SNS is based on interpersonal relationship, and SNS is an important platform for interpersonal communication (Diakopoulos and Naaman, 2011). SNS is based on a complex interpersonal network, and so organizations can seek value from consumers and other stakeholders through it (Abeza et al., 2020). Reasonable use of social media can promote interaction between consumers and enterprises thereby increasing the trust of consumers in enterprises and their intention to buy products (Hajli, 2014). SNS marketing can promote brand humanization, enhance the product experience, and loyalty of the consumer to the brand (Williamson, 2010). SNS has become a new platform for advertising marketing and it provides brands with unique opportunities to cultivate relationships with consumers. Enterprises can publish products or brand information to large amounts of users on SNS. With the rapid development of SNS, marketers are paying attention to various SNS opportunities and are beginning to implement new SNS marketing methods at an unprecedented speed.

Busyness is the perception of an individual to internal stress, which is caused by a lack of time to complete valuable work and often leads to absent-mindedness (Thompson et al., 2008). The research on busyness is mainly focused on the time allocation decisions, that is, how individuals allocate their time in paid work, household work, or with leisure.

Festini et al. (2016) have stated that the busyness of a person is related to the amount of working hours, speed and meaning, etc. A study by Gershuny (2005) showed that individuals who allocate more time to work are often considered to be busier. On the contrary, individuals who allocate more time to hobbies and interests are often considered to be more at leisure. Frequent SNS posting users are defined as those who frequently post Moments, Weibo, etc. (Hajli, 2014). Based on the above discussions, social media is a tool for information communication and entertainment. Frequent SNS posting users will be considered to spend more time on leisure and have plenty of leisure time.

Since the beginning of the last century, research on limited resources has appeared to have gradually developed into an important basic theory of economics research. Western economists believe that economics arises from the scarcity of objective existence and the need for choice. Behind this definition, it implies that the important idea of economics is that, the goods are scarce, and so the society must use resources effectively (Zhou, 2004). Kahneman (1973) believed that if resources and capacity are limited these resources can be flexibly allocated to complete multitasks or even do different things at the same time, but the premise of completing the task is that the required resources and capacity do not exceed the available resources and capacity. The resource of time as a kind of “virtual item” is the same for everyone, but the allocation of time by individuals is different. In other words, everyone can choose different ways of time consumption, such as work, leisure, housework, etc. Individuals who spend more time on social media and less time at work are considered at leisure (Gershuny, 2005; Bellezza et al., 2017; Schulte et al., 2017).

### Perceived Busyness and Social Status

Social status refers to the positions individuals or nuclear families occupy in the status structure of a given society, which is composed of education, occupation, sex, and marital status (Hollingshead, 1975; Bornstein et al., 2003; Adams and Weakliem, 2011). Some scholars have shown that individuals with high social status have higher initiative and stronger motivation. High initiative also makes people of high social status more resolute and courageous. Also, resources in social networks would contribute to the goal attainment of people and would improve the quality of life of an individual (Li et al., 2018). They have a higher action orientation than ordinary people and are less disturbed by the outside world. However, individuals with low social status are more likely to be influenced by the outside world and would often blindly follow the crowd (Davis, 1992; Galinsky et al., 2008; Brooks, 2010; Zhao et al., 2019). The actions of individuals with high social status are more oriented, and individuals with high social status can also guide the behavior habits of other individuals (Grewal and Stephen, 2019). Individuals with higher social status were less satisfied with unfair treatment than their lower status counterparts, supporting the perspective that high social status individuals feel entitled to more than low-status individuals (Hu et al., 2016). Compared with the research on the consequences of social status, the discussion on the antecedents of social status can be regarded as a new research topic. It is only in recent years that some scholars begin to devote themselves to this field. The private and subjective dimension of religion matters for well-being in China by helping adherents have an improved sense of social status relative to the non-religious (Chen and Williams, 2016). On Chinese social media platforms such as WeChat and QQ, Yang et al. (2017) found that SNSs use behaviors (i.e., selfies, status update, social feedback, and privacy control) that could also promote the attitude of individuals (i.e., desire for continuous attention and admiration of others). It is worth noting that studies have indicated that perceived busyness, education level, social class, religion, racism, and SNSs use behaviors as antecedents of social status (Thompson and Subich, 2011; Chen and Williams, 2016; Huang et al., 2016; Bellezza et al., 2017; Yang et al., 2017). The studies on the antecedents of social status are not large enough in number but influential.

Bellezza et al. (2017) discovered an alternative kind of conspicuous consumption that operated by shifting the focus from the preciousness and scarcity of goods to the preciousness and scarcity of individuals. Research has demonstrated that people who spend more time at work are considered to have higher social status in the United States where the perceived social mobility is high, but in a country with low perceived social mobility such as Italy, the conclusion is the opposite. In the study of perceived social status, the samples from the United States and Italy come to the opposite conclusion which further enlightened us to think about cultural differences. Social mobility refers to the change that moves people up and down the status ladder. Davidai and Gilovich (2015) put forward the concept of perceived social mobility from the perspective of individual subjectivity, which refers to the degree to which a person perceives that he can rise on the economic ladder in society. Perceived social mobility is reflected in a fair social system. People believe that they have the will to pursue opportunities and improve their social status through hard work (Bjørnskov et al., 2013). Social mobility is related to economic mobility, power mobility, consumption mobility, and status mobility. Low perceived mobility means low social economic mobility and low income (Ning, 2014). Under high social mobility, people at the bottom of the society will seize the opportunity to flow to the upper society; under low social mobility, people at the bottom of the society lack the chance to struggle for it (Xu and Liu, 2011). Social mobility is fundamental in American culture (Bellezza et al., 2017). Some studies have measured the social class of individuals by evaluating the availability of educational resources and have found that Americans believe that everyone has the opportunity to succeed through hard work (Kraus et al., 2010; Kraus and Tan, 2015). However, the Chinese people prefer to move to the upper class through direct inheritance (family wealth) or ties of homophily (social connection) (Li et al., 2015). The weakening of social mobility has become a critical problem when the Chinese social class is increasingly solidified and the people at the bottom of the society feel that they lack the opportunity to move to the upper class (Li and Zhou, 2014; Ning, 2014). The popularization of higher education has generally enhanced the extent of social equity and equality in Chinese. Nonetheless, as the return on education have flattened out recently, the social mobility has slowed down (Mok and Wu, 2016). In the case of low perceived mobility level in China, individuals who provide more working hours are more likely to be under greater life pressure. Correspondingly, they must provide more working hours to meet material needs.

First, we posit individuals who spend more time on social media and less time at work are considered leisure. Specific to social status, we propose that the more leisure people are the higher social status they are considered to possess. Although the relationship between frequent SNS posting and social status has not been tested directly, existing studies have found that the causal chain method, as the experimental psychological method, directly manipulates mediating variables and provides stronger causal evidence for mediating effects (Kim and Gal, 2014; McFerran and Argo, 2014; Han et al., 2016). In conclusion, we put forward hypothesis 1 and hypothesis 2:

***H1: More frequent SNS posting leads to higher status perceptions among Chinese consumers.***

***H2: This effect was mediated by perceived busyness, with consumers believing that people who post more frequently on SNS are less busy and therefore have higher social status.***

To test the hypotheses, we designed and conducted three empirical studies. Study 1 used secondary data to preliminarily verify the positive correlation between SNS posting frequency and perceived social status. The goal of Study 1 was to verify the basic effect of this study. After the basic effect has been verified, the underlying mechanism of the effect would be tested. Based on the increasing use of SNS, we considered the problems of how the posting frequency of WeChat Moment affects perceived busyness and how the perceived busyness affects this basic effect. Study 2 aimed to use the method of laboratory experiments to test H1 and H2. Studies 2A and 2B adopted the causal chain method to test the underlying mechanism of the effect. Specifically, Study 2A examined the relationship between the posting frequency of WeChat Moment and perceived busyness, followed by the manipulation of the mediating variables to further explore whether the differences in perceived busyness affect perceived social status and to provide direct support for the whole causal chain.

## Study 1

The main purpose of Study 1 is to establish the correlation between SNS posting frequency and perceived social status and to provide preliminary evidence for the basic hypothesis of the study.

### Measures

Study 1 takes universities in China as the object and analyzes the relationship between the frequency of publishing articles on their official accounts and the ranking of these schools. Specifically, we adopted the top 90 universities as the data of perceived social status, which comes from the top 600 list of the universities of China in 2020 released by Wu Shulian, leader of the research group of China Institute of Management Science in May 2020. We collected the number of official accounts issued by the official account platform of the school during the week from December 10, 2020 to December 16, 2020, and we used this data to measure the SNS posting frequency. After collecting the data, we analyzed the relationship between the ranking of the school and the official account posting frequency.

### Results

As shown in Table 1, the results of the correlation analysis initially supported the hypothesis of this article. Specifically, there is a significant negative correlation between the ranking of the school and the official account posting frequency (*r* = −0.28, *p* < 0.01).

**TABLE 1:** Results of descriptive statistics and correlation analysis (*N* = 90).

	***M***	***SD***	
The national ranking	45.50	26.13	
SNS weekly posting frequency	6.38	1.82	−0.28**

**p < 0.01; n = 90.

In other words, the top-ranked schools update their official accounts more frequently than the lower-ranked schools, that is, schools with higher social influence are more active on social media.

### Discussion

Study 1 used secondary data to preliminarily verify the positive correlation between SNS posting frequency and perceived social status. However, this only provides a small amount of support for the effects of SNS posting frequency and perceived social status. In addition, correlations cannot be an argument for causality. Therefore, we will use an experimental approach to further test this effect in the following study.

## Study 2A

With the causal chain method, we first manipulated the independent variable to examine the causal relationship between the independent and mediating variable, and then manipulated the mediator to test the relationship between the mediator and the dependent variable. These two steps, therefore, provide strong evidence for the entire causal chain from the independent variable to the dependent variable through mediation (Spencer et al., 2005). The causal chain method is also widely used in consumer research (e.g., Kim and Gal, 2014; McFerran and Argo, 2014; Han et al., 2016). Accordingly, Study 2A manipulates the frequency of WeChat Moments posting and tests its effect on perceived busyness, and Study 2B manipulates perceived busyness and explores whether it affects perceived social status.

### Participants and Procedure

Study 2A uses the method of laboratory experiments. We recruited 98 participants from the subject pool of a large public university in China. They would get extra credits for participating in experimental research. The number of men was 22 (22.4%) and women was 76 (77.6%). Before the experiment began, we assured him/her of anonymity.

**FIGURE 1:**
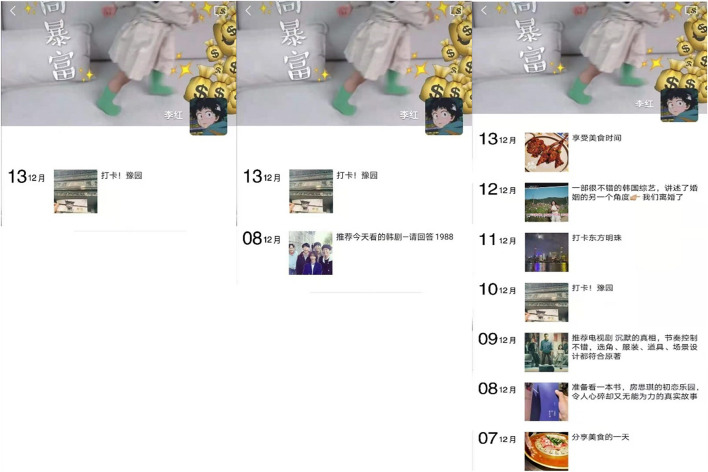
Pictures with different WeChat Moment posting frequency.

Study 2A adopted one factor between-subjects design (WeChat Moments posting frequency: 1 time vs. 2 times vs. 7 times). Participants were asked to read information about a virtual character Li Hong, and were shown with the content of Li Hong’s Moments in the past 7 days. We randomly assigned participants to one of the three conditions, such as post one WeChat Moment every week, post two WeChat Moments every week, post seven WeChat Moments every week. In order to control the potential confounding variables, the content of the WeChat Moment in the provided pictures is similar (all the content of the WeChat Moment is about life; as shown in Figure 1), the only difference is the WeChat Moment posting frequency. Next, all the participants were required to respond to the following items.

#### Items for Manipulation Check

Manipulation checks measured WeChat Moment posting frequency. In particular, all the subjects were asked about “according to the WeChat Moment in the past 7 days, what do you think of the number of WeChat Moment she sent?” (1 = the quantity is very small; 5 = the quantity is more).

#### Items for Busyness

We asked the subjects to evaluate Li Hong’s busyness according to the content of the picture. Participants answered the following two questions (Bellezza et al., 2017): “Li Hong has been working for a long time” “Li Hong is very busy” (1 = strongly disagree; 5 = strongly agree). We averaged the scores of the two questions (α = 0.78) as the dependent variable. Cronbach’s of those two items was 0.78.

### Results

#### Manipulation Checks

The Moments posting frequency is the dependent variable. One-way ANOVA was adopted in this experiment. The results showed that the frequency of Posting on Moments had a significant effect [*F*(2, 71) = 26.93, *p* < 0.01]. The more frequently Li Hong updated the Moments in a week, the subjects felt that the more Moments she posted (*M*_*one WeChat Moment*_ = 2.42, *SD*_*one WeChat Moment*_ = 1.14; *M_*two WeChat Moment*_* = 3.04, *SD_*two WeChat Moment*_* = 0.98; *M*_*seven WeChat Moment*_ = 4.40, *SD_*seven* WeChat Moment_* = 0.77).

The perceived busyness is the dependent variable. One-way ANOVA was adopted in this experiment. Study 2A explored whether the differences in WeChat Moment posting frequency affected perceived busyness, which means to compare the difference of perceived busyness when the WeChat Moment posting frequency is one, two, and seven. The results showed that the WeChat Moment posting frequency had a significant impact [*F*(2, 71) = 6.78, *p* < 0.05]. Specifically, frequency posting WeChat Moment would mean a higher level of leisure (*M*_*one WeChat Moment*_ = 2.83, *SD*_*one WeChat Moment*_ = 0.76; *M*_*two WeChat Moment*_ = 2.68, *SD*_*two WeChat Moment*_ = 0.69; *M*_*seven WeChat Moment*_ = 2.18, *SD*_*seven WeChat Moment*_ = 0.59). The result indicates that people who frequently post WeChat in a week are considered to be more at leisure and less busy. On the contrary, people who have infrequent WeChat postings in a week are considered to be busier and at less leisure.

### Discussion

Study 2A verified the relationship between the WeChat Moment posting frequency and perceived busyness through a laboratory experiment. In the following research, we will improve the discussion of the intermediary mechanism to supplement the entire chain of causality based on research 2A to verify the mediation mechanism.

## Study 2B

### Participants and Procedure

Study 2B uses the method of laboratory experiments. We recruited 218 participants from the subject pool of a large public university in China. They could get extra credits for participating in experimental research. The number of men was 45 (20.6%) and women was 173 (79.4%). Before the experiment began, we assured him/her of anonymity. The research adopted one factor between-subjects design (Conditions: busyness vs. leisure). We randomly assigned participants to one of the two conditions, namely busyness and leisure (Bellezza et al., 2017). Participants were asked to read information about a virtual character Li Hong’s life. The information in the busyness condition is “Li Hong has been working all week, spending 10 min to eat at noon every day.” While the information in the leisure condition is “Li Hong hardly works this week and enjoys a long noon break every day.” Next, all the participants were required to respond to the following items.

#### Items for Manipulation Check

Lastly, manipulation checks measured Sally’s level of busyness, Specifically, they were asked: “Li Hong spends many hours at work;” “Li Hong is busy” (1 = strongly disagree, 5 = strongly agree). We averaged the scores of the two questions (α = 0.84) as the mediating variables (perceived busyness).

#### Items for Perceived Social Status

Subsequently, we measured perceived social status using two distinct measures. A primary measure of status was developed based on previous status definitions (Veblen and Galbraith, 1973; Bourdieu, 1984; Scott et al., 2013) to include both social status and financial resources (wealth and income). Specifically, participants answered the following two questions: “how would you rank the financial resources, social influence and state of the individual described” “how would you rank the social status of the individual described.” (1 = low social status; 5 = high social status). We averaged the scores of the two questions (α = 0.94) as the dependent variable (social state).

**FIGURE 2:**
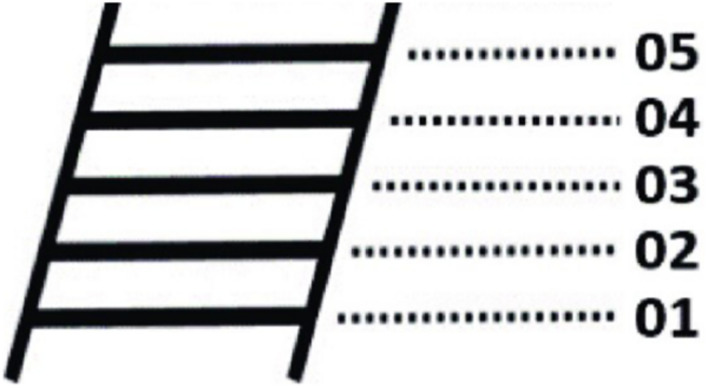
The MacArthur Scale of Subjective Socioeconomic Status.

We adapted the widely used MacArthur scale of subjective socioeconomic status (Adler et al., 2000; Anderson et al., 2012) to assess the status of a third party. The measure consists of a drawing of a ladder with 10 rungs representing where people stand in society in terms of money, status, and influence (5 represents people at the top of society and 1 represents people at the bottom of society, as shown in Figure 2). The participants were told that the people at the top of the ladder were at the top of the society and they had the best living conditions, the highest income, the highest education level, and the most decent work. The people at the bottom of the ladder are at the bottom of the society with the worst living conditions, lowest income, the lowest education level, and the least decent work. Participants were instructed to pick the rung where they would place Li Hong (Adler et al., 2000; Anderson et al., 2012).

### Results

Manipulation checks. Perceived busyness is the dependent variable. One-way ANOVA was adopted in this experiment. The results showed that the manipulation of busyness had a significant effect [*F*(1, 216) = 182.15, *p* < 0.001]. Compared with the participants in the leisure condition (*M*_*leisure*_ = 2.52, *SD*_*leisure*_ = 0.91), participants in the busy condition (*M*_*busy*_ = 4.12, *SD_*busy*_* = 0.84) perceived Li Hong as more busyness. The manipulation of the level of busyness is effective.

The social status is the dependent variable. One-way ANOVA was adopted in this experiment. The results are consistent with our expectations. Compared with participants in the busy condition (*M_*busy*_* = 2.76, *SD*_*busy*_ = 0.71), participants in the leisure condition (*M*_*leisure*_ = 3.37, *SD*_*leisure*_ = 0.75) perceived Li Hong as higher in social status [*F*(1, 216) = 37.90, *p* < 0.001]. They placed her on a higher rung on the socioeconomic status ladder (*M_*busy*_* = 2.79, *SD*_*busy*_ = 0.76; *M_*leisure*_* = 3.39, *SD_*leisure*_* = 0.69).

### Discussion

Study 2B directly manipulates the perceived busyness, and the results show how the differences in perceived busyness affect social status perceptions, which further verifies the mediating role of perceived busyness. These hypotheses received convergent support in a set of three studies. In conclusion, Studies 2A and 2B provide strong support for the mechanism of conspicuous behavior on SNS. In this paper, hypothesis 1 and hypothesis 2 are verified by three experiments.

## General Discussion

### Research Findings

This research systematically introduces the relationship between SNS posting frequency and perceived social status. Firstly, the target of study 1 is to establish the correlation between SNS posting frequency and perceived social status so as to provide preliminary evidence for the basic hypothesis of the study. This research uses secondary data to preliminarily verify the correlation between SNS posting frequency and perceived social status. Study 1 shows that schools with higher social status update their official accounts more frequently, revealing the positive correlation between SNS posting frequency and perceived social status. Correlations cannot be an argument for causality. Therefore, in Study 2, the research uses an experimental approach to further test this effect. Studies 2A and 2B adopt the causal chain method to test the underlying mechanism of the effect, and to provide causal evidence for the entire relationship chain. Research 2A uses WeChat Moment posting frequency as the symbol of SNS posting frequency. The results of study 2A shows that people who post WeChat more frequently in a week are considered to be more at leisure and less busy. On the contrary, people who have lower frequency posting on WeChat in a week are considered to be busier and less at leisure. The results of Study 2B indicates that the busier people are considered to have the lower social status. Conversely, the more leisure people are considered to have the higher social status. The results of Studies 2A and 2B provide directly support for the causal chain of mediating effect. In general, the research systematically verifies the relationship between SNS posting frequency and perceived social status, and further verifies the internal psychological mechanism of this basic effect. The research proves that this effect is universal (whether it is correlation or causal relationship).

### Theoretical Contributions

Nowadays more and more consumers show status in more implicit ways. The research has indicated that consumers will judge the social status of users based on the SNS posting frequency. In addition, the research tests the underlying mechanism of the effect. Therefore, the first theoretical contribution of this paper is the literature on SNS. Being different from previous researches on online consumer behaviors (e.g., Williamson, 2010; Khan, 2017; Mieczkowski et al., 2020), this research explores the impact of SNS on perceived social status. Current research on online consumer behavior mainly focuses on Western consumers (Williamson, 2010; Hajli, 2014; Abeza et al., 2017), whereas this research takes the impact of cultural differences into consideration. Different from previous researches which focus on the consequences of social status (e.g., Davis, 1992; Galinsky et al., 2008; Brooks, 2010), this research is consistent with the latest research directions (Thompson and Subich, 2011; Bellezza et al., 2017; Yang et al., 2017). The research is dedicated to understanding the antecedents of social status. By verifying the correlation between SNS posting frequency and perceived social status, this study further enriches and provides further evidence for this series of studies.

The research also makes a theoretical contribution to the literature of conspicuous consumption. Existing literature on consumer behaviors mainly concentrates on explicit conspicuous signals (e.g., Ordabayeva and Chandon, 2011; Wang et al., 2015; Gao et al., 2016); The research investigated the subtle signals of status on SNS. Consumers purchase products with obvious conspicuous signals to improve their social status, but some subtle signals can also play the same role (e.g., Dubois et al., 2012; Shao, 2020; Shen, 2020; Valsesia et al., 2020). This research further tests how to make conspicuous consumption in SNS, which comes to a conclusion that people who have frequent SNS posting are considered to have higher social status.

### Substantive Contributions

In addition to significant theoretical contributions, this study also has direct instructive value for consumers, enterprises, policy makers, etc. Firstly, this research reveals the psychological mechanism of the dazzling wealth of consumers in SNS. If someone wants to show his social status, in addition to buying general luxury goods, improving the activity of SNS is also an effective way. Secondly, consumers are more willing to believe that individuals with frequent SNS posting are considered to have high social status and can play the role of consumer purchase orientation.

Research shows that the content generated by users on SNS is able to guide the behavior of consumers, which reduces the impact of traditional marketing. Companies should not only rely on traditional marketing promotion, but also participate more in SNS activities, and further use SNS to enhance corporate brand image and promote interaction with potential consumers.

Internet is an immaterial zone beyond time and space limitation, which emerged with the development of the information and technology industry. Based on the advent of Internet technology, various forms of social media have aroused and become cyber-communities that corresponded with the living habits of young consumers. Generally speaking, a cyber-community constructed by social media can be described as follows: (1) low access threshold, (2) low authority, and (3) huge user stock and traffic. Therefore, the findings of the research also call for the government to strengthen the guidance of information on social media.

### Research Limitations and Future Research Directions

It is important to acknowledge that though we strive to be rigorous and detailed in each step, there are still limitations due to several factors, and these limitations point to the future research direction which is described below.

Laboratory experimental method was used in this study. Since the experimental sample is not randomly sampled and the subjects are not representative enough of all the consumers, the external validity of the study may be reduced and the inferential ability may be weaker. For example, it’s probable that some individuals do not browse SNS in the recruitment objects. In the future research, we need to screen out those who have relevant browsing experience in advance to increase the external validity. Besides, other possible confounders remain unchecked. For example, the posting content may change the social status perception of subjects. Specifically, frequent posts about travel and eating-out experiences may send a signal that the person posting them has the spare cash to do so. Future research should examine whether the money-spending signals underlying these activities confound our causal chain.

The experimental site is similar to the real SNS, but differences still exist. The location of the experiment is not exactly the same as the place where the subjects usually visit SNS, which may also lead to some errors. To improve the experiment, the overarching design should be as realistic as possible, and the process of the experiment ought to be as simple and clear as possible to avoid making the participants feel that they are doing the experiment instead of immersing themselves in the simulated network. The future research can adopt the method of field experiment to test the hypothesis in the “real” situation and verify the causal relationship.

This research verifies the basic effect relationship between SNS posting frequency and perceived social status, and considers the psychological process of this effect as perceived busyness. In the future research, we can broaden the research in this field and look for possible boundary conditions. For example, environmental variables including social environment and physical environment might play a moderating role.

Additionally, though our focus was on the subtle signals of status for Chinese consumers on SNS, another interesting question that stems from Study 2B is that whether people with more leisure time have higher social status. It would also be interesting to explore whether people with high social status play a leading role in social media, which means they act as opinion leaders according to the KOL theory (Moynihan, 2008). For instance, do individuals with higher social status have greater SNS influence, i.e., a large number of followers, huge appeal, and so on? These possibilities await further investigations.

## Data Availability Statement

The raw data supporting the conclusions of this article will be made available by the authors, without undue reservation.

## Ethics Statement

The studies involving human participants were reviewed and approved by the research ethics board of Shanghai University of International Business and Economics. Written informed consent for participation was not required for this study in accordance with the national legislation and the institutional requirements.

## Author Contributions

WW designed the studies and made a significant revision to the earlier version of the manuscript. TS assisted in research and data collection and wrote the manuscript. YY collected and analyzed the data. SF and JS revised the manuscript. ZY designed the studies and revised the manuscript. All authors contributed to the article and approved the submitted version.

## Conflict of Interest

The authors declare that the research was conducted in the absence of any commercial or financial relationships that could be construed as a potential conflict of interest.

## Publisher’s Note

All claims expressed in this article are solely those of the authors and do not necessarily represent those of their affiliated organizations, or those of the publisher, the editors and the reviewers. Any product that may be evaluated in this article, or claim that may be made by its manufacturer, is not guaranteed or endorsed by the publisher.
